# Characterization of abscopal effects of intratumoral electroporation-mediated IL-12 gene therapy

**DOI:** 10.1038/s41434-018-0044-5

**Published:** 2018-10-15

**Authors:** Anandaroop Mukhopadhyay, Jocelyn Wright, Shawna Shirley, David A. Canton, Christoph Burkart, Richard J. Connolly, Jean S. Campbell, Robert H. Pierce

**Affiliations:** 1 OncoSec Medical Incorporated, 3565 General Atomics Court #100, San Diego, CA 92121 USA; 2 Fred Hutchinson Cancer Center, 1100 Fairview Avenue N, Seattle, WA 98109 USA

**Keywords:** Tumour immunology, Immunotherapy

## Abstract

Intratumoral electroporation-mediated IL-12 gene therapy (IT-pIL12/EP) has been shown to be safe and effective in clinical trials, demonstrating systemic antitumor effects with local delivery of this potent cytokine. We recently optimized our IL-12 gene delivery platform to increase transgene expression and efficacy in preclinical models. Here we analyze the immunological changes induced with the new IT-pIL12/EP platform in both electroporated and distant, non-electroporated lesions. IT-pIL12/EP-treated tumors demonstrated rapid induction of IL-12-regulated pathways, as well as other cytokines and chemokines pathways, and upregulation of antigen presentation machinery. The distant tumors showed an increase in infiltrating lymphocytes and gene expression changes indicative of a de novo immune response in these untreated lesions. Flow cytometric analyses revealed a KLRG1^hi^ CD8^+^ effector T-cell population uniquely present in mice treated with IT-pIL12/EP. Despite being highly activated, this population expressed diminished levels of PD-1 when re-exposed to antigen in the PD-L1-rich tumor. Other T-cell exhaustion markers appeared to be downregulated in concert, suggesting an orchestrated “armoring” of these effector T cells against T-cell checkpoints when primed in the presence of IL-12 in situ. These cells may represent an important mechanism by which local IL-12 gene therapy can induce a systemic antitumor immune response without the associated toxicity of systemic IL-12 exposure.

## Introduction

Interleukin 12 (IL-12) is pleotropic inflammatory cytokine, which links innate and adaptive immunity and drives Th1/Tc1 cell-mediated immune responses. IL-12, therefore, would be a good candidate for increasing tumor immunogenicity. However, systemic administration of recombinant IL-12 protein [1–3] or IL-12-expressing adoptive cell therapies [4] have led to severe immune-related toxicities in patients. As an alternative, intratumoral (IT) gene therapy with plasmids expressing IL-12 has been tested both in experimental mouse models and in the clinic. As a monotherapy in advanced melanoma, IT electroporation-mediated transfection of a plasmid encoding human IL-12 yielded a 33% best overall response rate, with 50% of patients showing regression of untreated lesions, without any reported systemic drug-related toxicity [5, 6].

In mouse models, previous studies have shown that IL-12 introduced IT reduces tumor burden and prolongs survival [7–14]. In addition to reducing growth of treated tumors, IT IL-12 treatment induces a memory response, which protects mice from tumor re-challenge [7, 8, 11, 12], as well as reducing growth of metastases [8, 9] and untreated distant tumors [7, 12]. Effects of IT treatment with IL-12 on IT immunogenicity include local influx of CD8^+^ T cells, production of interferon-γ (IFNγ), and resultant increase in major histocompatibility complex (MHC) class I expression in treated tumors [7, 13]. General increase in infiltrating lymphocytes and reduction in tumor vascularity have also been noted in treated lesions [8]. Dissemination of tumor antigen-associated (TAA) CD8^+^ T cells with IT IL-12 have been reported and depletion of CD8^+^ T cells diminished IL-12 antitumor effects, whereas CD4^+^ or NK depletion had a modest effect [7]. These depletion studies indicate that IT IL-12 therapy-mediated tumor immunity is mediated, in large part by CD8^+^ T cells, and suggest that IT IL-12 treatment may function as an in situ vaccine through the induction of cell death-mediated antigen release in a pro-inflammatory, strongly Th1-biased tumor microenvironment (TME) [15].

Based on previous clinical and preclinical studies and our recent work demonstrating a dose-dependent effect of IT-pIL12/EP, we optimized our therapy to maximize transgene expression through modifications to the plasmid and electroporation parameters, resulting in significant systemic tumor growth control with a single treatment [16]. The IL-12 encoding plasmid contained the p35 and p40 subunits of murine IL-12 expressed from a polycistronic message that utilizes the picornovirus exon-skipping motif, P2A. In addition to a novel plasmid design, the new IT-pIL12P2A/EP platform uses a reduced electric field strength and increased pulse duration to enhance transfection efficiency [17]. Expression of transgenes were detected in nearly 10% of tumor cells, approaching the efficiency of viral transduction methods [16]. Robust expression of IL-12p70 protein was detected in the electroporated lesion (EL) but remained low in serum [16].

In this study, we explore the mechanism of action of IT-pIL12P2A/EP using a two-tumor contralateral model, created by flank injection of B16F10 melanoma cells into syngeneic C57BL/6 mice with particular emphasis on understanding “abscopal” effects in distant non-treated lesions (contralateral tumor). We provide evidence that IT IL-12 treatment leads to induction of IL-12-regulated genes, other cytokine and chemokines pathways, as well as genes for enhanced antigen processing and presentation in the treated tumor. These localized IL-12-mediated effects led to the generation of systemic tumor immune responses, including a surge of KLRG1^hi^CD8^+^ effector T cells detected in the spleen and in contralateral, non-treated tumor-infiltrating lymphocytes (TILs). Interestingly, this systemic immune response is accompanied by induction of IFNγ-mediated adaptive immune resistance markers, despite significant growth control of the distant, contralateral tumor. The IL-12-driven KLRG1^hi^CD8^+^ effector cells detected in the Programmed death-ligand 1 (PD-L1)-rich contralateral TME had decreased expression of PD-1, as well as other cell surface exhaustion markers as compared with those in control animals. Our results suggest that CD8 T cells primed in the IL-12-treated TME may be, at least transiently, “armored” against multiple T-cell checkpoints, contributing to their fitness as antitumor cytotoxic T lymphocyte (CTL) and may represent a prominent mechanism by which local IT IL-12 gene therapy can deliver a safe and effective abscopal response.

## Results

**Fig. 1:**
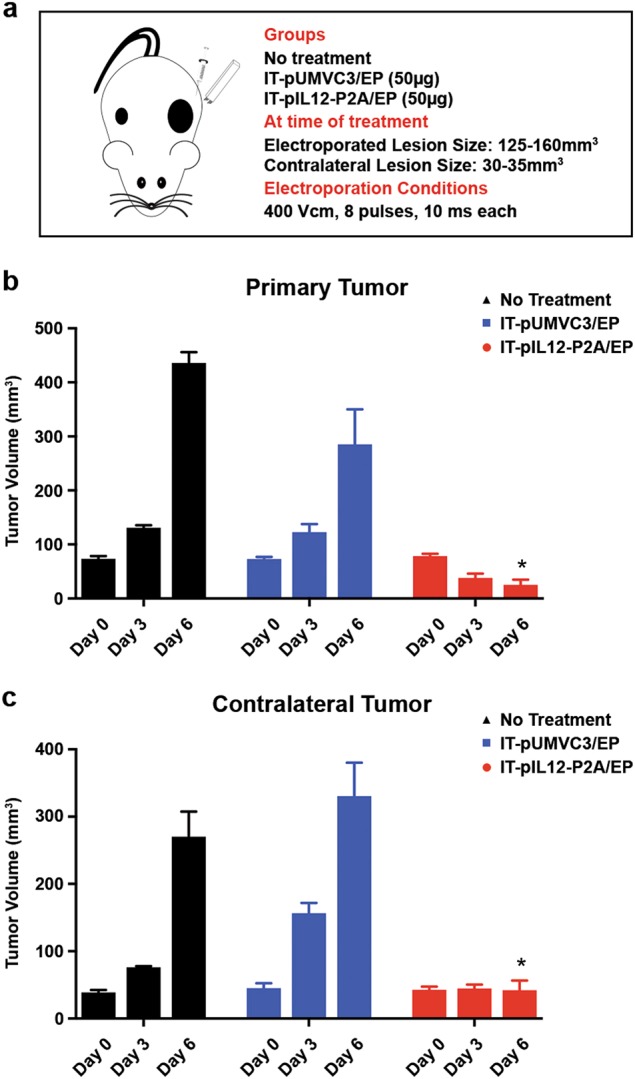
IT-pIL12/EP led to tumor regression in a B16F10 contralateral tumor model. **a** Schematic of a two-tumor contralateral model; B16F10 or B16F10-OVA cells were implanted on both flanks of C57Bl/6J 6- to 8-week-old female mice at different densities (1 million cells on left flank and 0.25 million cells on the right). Tumor (125-160 mm^3^) on left flank was electroporated, whereas the tumor (30–35 mm^3^) on the right flank (distant contralateral lesion) was left untreated. Treatment groups include IT injection of 50 μg of empty vector plasmid control (pUMVC3) or 50 μg of pIL12-P2A plasmid or untreated (no DNA or EP). **b** Growth of primary (electroporated) and **c** contralateral B16F10 lesions after IT-pIL12/EP (red) and IT-pUMVC3/EP (blue) and no treatment (black) are shown. Six days post treatment for both electroporated and contralateral tumors (*n* = 5; statistical significance determined using two-way ANOVA with Bonferroni correction, *p < 0.0001). IT-EP treatment was performed once on day 0. Measurements for day 0 (pretreatment), and day 3 and 6 (post treatment) are shown. At 6 days post treatment, both electroporated and contralateral tumors from the IT-pIL12/EP cohort were significantly smaller (*n* = 5; statistical significance determined using two-way ANOVA with Bonferroni correction, *p < 0.0001) (color figure online)

To study the mechanisms of action of the optimized IT-pIL12/EP therapy on distant, untreated lesions, a two-tumor contralateral model previously described [16] was used. The poorly immunogenic B16F10 cell line (C57Bl/6J mice) was implanted in the subcutis on opposite flanks, on the left (1,000,000 cells) and on the right (250,000 cells). Ten days post implantation, left tumors were electroporated once after injection with pIL12P2A (IT-pIL12P2A/EP) or empty vector (IT-pUMVC3/EP). As a control, some tumor-bearing mice were left untreated. Mice receiving IT-pIL12/EP (IT-pIL12P2A/EP) demonstrated significantly reduced growth of the EL, as well as untreated lesions by day 6 as compared with control groups (Fig. 1). A slight growth delay in the EL was observed in the empty vector-treated mice as compared with untreated mice, however, no effect on the distant, contralateral  tumors was observed.

### Gene expression changes in the TME of IT-IL-12-EP-treated tumors

**Fig. 2:**
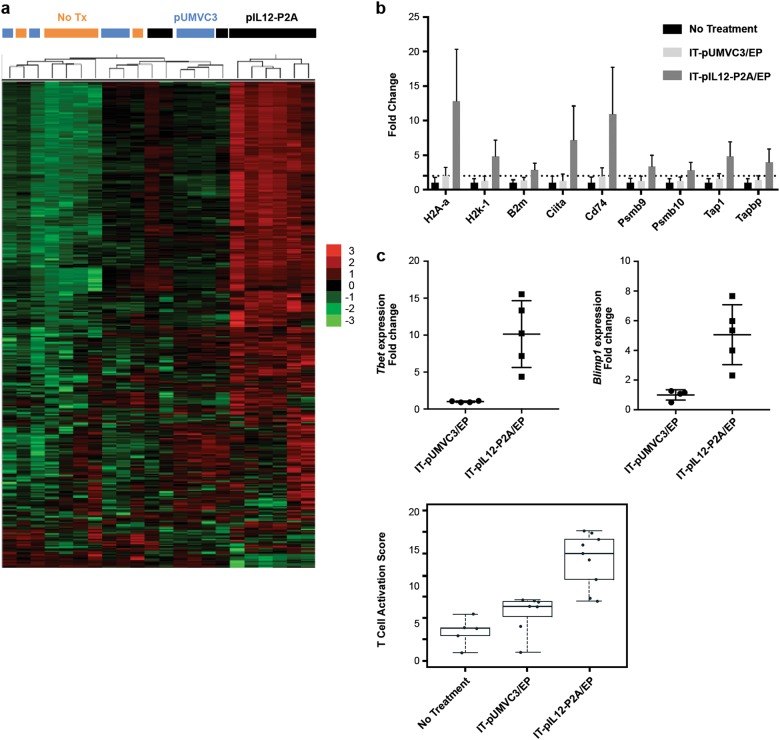
IT-pIL12/EP induced conversion to a pro-immunogenic microenvironment in treated tumors. **a** Gene expression changes 7 days post treatment in electroporated B16F10 lesions assessed by NanoString^®^ nCounter technology. Treatment groups were IT-EP of 50 μg of empty vector plasmid (pUMVC3) or of 50 μg of pIL12-P2A plasmid, as well as no treatment control mice. Hierarchical clustering and heat map (Z scores) representation of all genes. Significant clustering of 6/9 mice receiving pIL12-P2A (black bar) was observed. Untreated (no Tx, orange bar) and empty vector plasmid (pUMVC3, blue bar) treated samples did not cluster suggesting no significant difference between the groups (*n* = 9 for pIL12-P2A, *n* = 7 for pUMVC3, *n* = 6 for untreated). **b** Induction in expression of APM genes, expressed as a fold change over no treatment levels at 7 days post treatment as assessed by nCounter technology (all genes presented were statistically different from controls (untreated tumors); *p* < 0.05, Holm–Sidak method). The dotted line represents a twofold increase over untreated tumor controls. **c** Top panel: fold change in *Blimp1* and *Tbet* gene expression 7 days post treatment in mice receiving pIL12 (*n* = 5 for IL-12-P2A, black squares) compared with empty vector plasmid (*n* = 4 for pUMVC3, black circles). Statistical significance was calculated using *T-*test (*p* < 0.01). Bottom panel: enrichment of genes involved in T-cell activation 7 days post treatment represented as ‘Pathway scores’ determined by nSolver data analysis software. Pathway scores follow the assumptions of equal variance and normal distribution of t scores. The T-cell activation score in IL-12-P2A group (*n* = 9) was significantly higher than the pUMVC3 (*n* = 7) and untreated group (*n* = 6) (*p* < 0.0001, one-way ANOVA) (color figure online)

Seven days after EP, treated tumors were excised and total RNA was prepared from the tumor tissue. The transcriptional status of immune-related genes was assessed using the NanoString^®^ nCounter platform. Hierarchical agglomerative clustering of 561 immune-related genes showed robust clustering of mice receiving IT-pIL12/EP (6/9 mice; Fig. 2a) indicating a strong IL-12-mediated modulation of the transcriptional status of immune genes in the treated B16F10 TME. An increase in transcripts associated with T lymphocytes (*Cd3, Cd4, Cd8*), as well as myeloid-lineage cells (*Cd11b, Cd11c, Emr1, Cd14*; Figure S1b) in IT-pIL12/EP-treated tumors were seen. Treated tumors were also analyzed morphologically by hematoxylin and eosin (H&E) staining of formalin-fixed, paraffin-embedded sections at this time point, revealing extensive tissue necrosis, in association with IT hemorrhage and a marked pleomorphic inflammatory infiltrate, particularly pronounced at the margin of the tumor (Figure S1a). Focal, small areas of tumor necrosis were identified in tumors treated with IT-pUMVC3/EP, but the extent of necrosis and the presence of hemorrhage and an associated inflammatory response were far diminished in comparison.

Many of IL-12 pleotropic pro-inflammatory effects, in particular, the generation of downstream antitumor cytotoxic T-cell responses, have been shown to be dependent on the downstream production of IFN-γ [18, 19]. B16F10 tumors directly electroporated with pIL12 demonstrated a robust gene signature suggestive of IFN-γ activation as expected (Figure S1c). Given the role of IFN-γ in modulating genes involved in antigen processing and presentation machinery (APM), we investigated expression of transcripts associated with APM. IT-pIL12/EP led to a significant upregulation of multiple genes associated with antigen presentation on both MHC class I (*H2-Kb, Tap1, Tapbp, Psmb9/10, B2m*) and MHC class II (*H2-Aa, Ciita, Cd74*) receptors (Fig. 2b).

Two transcription factors, *Blimp-1* and *T-bet*, known to be regulated by IL-12 receptor signaling [20, 21], and important regulators of T-cell homeostasis, were induced 5-fold and 10-fold, respectively, in the treated TME with IT-pIL12/EP as compared with control mice (Fig. 2c). In addition, tumors electroporated with IL-12 were enriched for genes involved in T-cell activation as determined by pathway scores using nSolver analysis software (Fig. 2c). Pathway scores generated with nSolver also demonstrated an upregulation of genes involved in the Janus kinase/signal transducers and activators of transcription   (JAK/STAT) pathway specifically in the IT-IL-12-EP group at day 7 (data not shown). As activation of this pathway is a direct downstream event in IL-12 signaling [22], we investigated the kinetics of enrichment of this gene signature (JAK/STAT).

In a separate experiment, treated tumors were excised 72 h after treatment and a NanoString^®^ nCounter platform analysis was performed with the same immune-related gene panel. Significant clustering of IT-pIL12/EP-treated mice was observed (5/5 mice, data not shown). Genes involved in the JAK/STAT pathway were already significantly upregulated at 72 h in the IT-IL-12-EP group (Figure S2a). Furthermore, several immunostimulatory cytokines and chemokines involved in tumor inflammation were also upregulated in the IT-pIL12/EP group 72 h post EP (Figure S2b). Chemokines function in the recruitment of leukocytes important for both innate and adaptive immune responses. An upregulation of genes involved in activation of an innate response was observed in the IT-IL-12 EP groups at 72 h (Figure S2c) and remained high 7 days post EP (data not shown). An upregulation of other genes involved in inflammation were also observed early in tumors electroporated with IL-12 (Figure S2d). The striking upregulation of diverse inflammatory gene expression signatures (Fig. 2, S1, and S2) together with substantial tumor necrosis, hemorrhage and robust inflammatory infiltrate (Figures S3) illustrate that all of the components are present to suggest IT-pIL12P-EP functions as an in situ vaccine, leading to immunogenic tumor cell death and priming of a de novo adaptive immune response.

### IT-pIL12/EP increased tumor lymphocyte infiltration and markers for adaptive immune resistance in distant, contralateral lesions

**Fig. 3:**
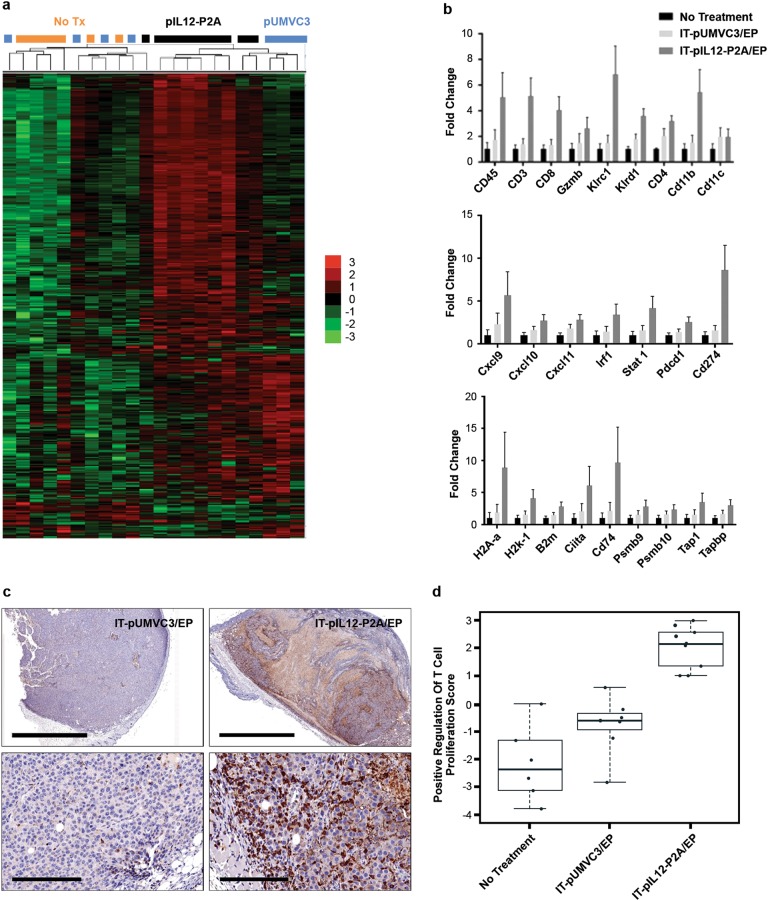
IT-pIL12/EP increased TIL and led to adaptive immune resistance markers in contralateral tumors. Gene expression changes 7 days post treatment in contralateral B16F10 lesions. **a** Hierarchical clustering and heat map (Z scores) representation of all genes is presented. Significant clustering of 6/9 mice receiving IT-pIL12-EP (black bar) was observed. Untreated (no Tx, orange bar) and empty vector plasmid (pUMVC3, blue bar) treated samples did not cluster respectively suggesting no significant difference between the groups (*n* = 9 for pIL12-P2A, *n* = 7 for pUMVC3, *n* = 6 for no Tx). **b** Induction in expression of markers for immune infiltrates (top panel), IFN-γ-responsive genes (middle panel) and APM-related genes (bottom panel), all expressed as a fold change compared with no treatment levels at 7 days post treatment as assessed by NanoString^®^ nCounter technology (all genes presented were statistically different from controls; *p* < 0.05, Holm–Sidak method). **c** Immunohistochemical stain of murine CD8 protein (brown) in contralateral tumors at day 7 post EP (left: IT-pUMVC3/EP, right: IT-IL-12-EP). Low power scale bar = 2000 μm, High power scale bar = 200 μm. **d** Enrichment of genes involved in proliferation of T cells 7 days post treatment represented as ‘Pathway scores’ determined by nSolver data analysis software (NanoString^®^). Pathway scores follow the assumptions of equal variance and normal distribution of *t* scores. The ‘positive regulation of T-cell proliferation score’ in IL-12-P2A group (*n* = 9) was significantly higher than the pUMVC3 (*n* = 7) and untreated group (*n* = 6) (*p* < 0.0001, one-way ANOVA) (color figure online)

IT-pIL12/EP slowed the growth of distant, contralateral lesions  significantly more than the empty vector group after a single treatment [16] (Fig. 1b). Seven days after treatment of tumors as described above, the contralateral tumors were excised and total RNA was prepared. Changes in gene expression were measured using the NanoString^®^ nCounter platform to investigate the mechanisms of growth inhibition of these distant, untreated lesions. Hierarchical agglomerative clustering of 561 immune-related genes from these cohorts showed that mice whose primary tumors received IT-pIL12/EP clustered together (4/5 mice**;** Fig. 3a), indicating perturbations in gene expression in distant lesions mediated indirectly by IL-12 priming in treated tumors. Furthermore, the contralateral lesions from mice treated with IT-pIL12/EP showed robust increases in transcripts associated with IFN-γ signaling, T-cell activation, as well as those associated with lymphocytes and myeloid-lineage cells (Fig. 3b). Histochemical (H&E) and immunohistochemical staining (anti-CD8a) of the distant, non-electroporated distant tumors demonstrated the presence of a pronounced CD8^+^ T-cell-rich inflammatory infiltrate in IT-pIL12/EP mice (Fig. 3c), which was relatively scant in control-treated mice, corroborating the observation of increased T cells inferred based on gene expression of *Cd8a, Cd3e* transcripts (shown in Fig. 3b*)*. In addition, genes that are known to positively regulate T-cell proliferation were also upregulated in IT-pIL12/EP mice (pathway score analysis, Fig. 3d) suggestive of a robust immune response. The induction of such a significant IFN-γ signal in the distant, non-electroporated tumors suggests that T cells are engaging their cognate antigen in the contralateral TME, leading to local T-cell-mediated IFN-γ production and downstream genes involved in adaptive immune resistance (e.g., PD-L1).

We previously demonstrated that IL-12p70 is detectable in IT-pIL12/EP-treated tumors but not significantly in serum using our new therapy platform [16]. To verify that the plasmid-derived IL-12 expression is not present in distant, contralateral lesions, we performed an enzyme-linked immunosorbent assay (ELISA) on tumor tissue with an antibody directed against a P2A epitope that remains on the p35 subunit of plasmid-derived IL-12 cytokine. The treated and distant untreated tumors from mice were harvested 48 h after IT-pIL12/EP and ELISA performed on the tissue lysates. P2A antigen was detected in the electroporated tumors but not in distant,  contralateral tumors (Figure S3) or in serum (data not shown), suggesting that IL-12p70-P2A protein translated from plasmids that were delivered by IT electroporation is largely confined to the treated tumor. Antitumor effects seen in distant contralateral lesions are therefore, likely mediated by changes in tumor immunity generated in the treated tumors or associated lymph nodes.

### IT-pIL12/EP led to systemic expansion of tumor-specific SIINFEKL tetramer^+^ CD8^+^ effector T cells

**Fig. 4:**
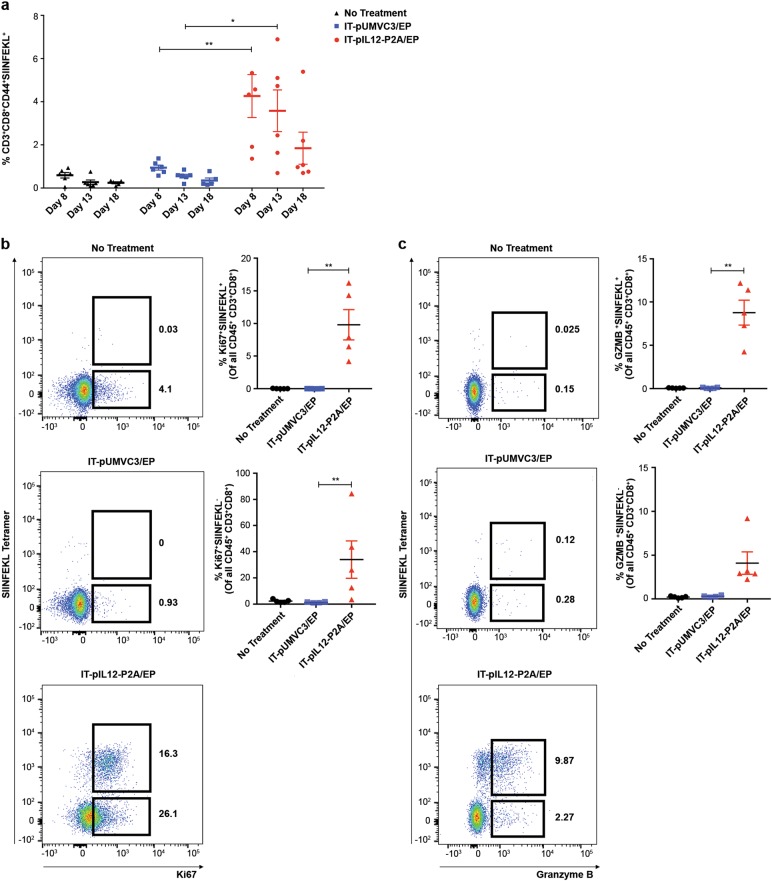
Time course of flow cytometric analysis of splenocytes from mice treated with a single dose of IT-pIL12/EP therapy; persistence of TAA-CD8^+^ effector cells and enhanced expression of proliferation and cytotoxic markers. **a** Splenocytes were isolated 8, 13 and 18 days post EP from B16-OVA tumor-bearing mice intratumorally electroporated with pIL12P2A (red), pUMVC3 (blue) or left untreated (black) and analyzed by flow cytometry. Scatter plots show mean ± SEM percentage of all LIVE splenocytes that were CD19^NEG^/NK1.1^NEG^/CD3^+^/CD8^+^/CD44^+^/SIINFEKL tetramer^+^ at each time point (*n* = 6 mice per cohort per time point; total *n* = 18 for each treatment group; **p < 0.01, *p < 0.05, individual *t*-test, Holm–Sidak method). Results presented were confirmed in two other independent electroporation experiments. **b** In a separate experiment, splenocytes were isolated 7 days post EP from B16-OVA tumor-bearing mice left untreated or intratumorally electroporated with pIL12-P2A or pUMVC3 control and analyzed by flow cytometry. Splenocytes were gated as follows prior to analysis: LIVE, CD19^NEG^NK1.1^NEG^/CD3^+^CD8^+^cells. Representative dot plots from one mouse per cohort show gating for Ki67^+^SIINFEKL^+^CD8^+^ and Ki67^+^SIINFEKL^NEG^CD8^+^ (left panels). Scatter plots show quantification (%) of Ki67^+^SIINFEKL^+^,Ki67^+^SIINFEKL^NEG^ from all mice (right panels, *n* = 5 per cohort **p-value = 0.009, one-way ANOVA, Kruskal–Wallis test). **c** Splenocytes isolated 7 days post EP from B16-OVA tumor-bearing mice were gated as described in (**b**) and analyzed by flow cytometry. Representative dot plots show gating for granzyme B^+^SIINFEKL^+^CD8^+^ T and granzyme B^+^SIINFEKL^NEG^CD8^+^ T cells from one mouse per cohort (left panels). Scatter plots show quantification (%) of granzyme B^+^ SIINFEKL^+^ and granzyme B^+^ SIINFEKL^NEG^ CD8^+^ T cells from all mice (right panels, *n* = 5 per cohort *p-value = 0.021, one-way ANOVA, Kruskal–Wallis test). Results presented were repeated in two independent electroporation experiments (color figure online)

To monitor IT-pIL12/EP generated tumor antigen-specific CD8 T cells, both systemically and in the contralateral TIL, we employed a B16F10 variant cell line, expressing the well-defined OVA antigen, containing the K^b^-restricted CD8 epitope, SIINFEKL. IT-pIL12/EP controlled the growth of both electroporated and non-electroporated B16F10-OVA tumors similar to what was seen in the B16F10 model (data not shown). To quantify the TAA CD8 (TAA-CD8^+^) T cells, splenic lymphocytes were isolated from mice bearing B16F10-OVA tumors 8, 13, and 18 days post EP, stained with SIINFEKL tetramer and analyzed by flow cytometry. Enrichment of activated CD44^+^, SIINFEKL^+^CD8^+^ T cells was observed in the IT-pIL12/EP-treated group compared with IT-pUMVC3/EP-treated and untreated mice (~70-fold; Fig. 4a), in agreement with a previous study showing IT-IL-12 induced dissemination of TAA-CD8 + T cells [7]. When examined over the time course, the elevated TAA-CD8^+^ T-cell population persisted in the spleens of IL-12-treated mice for 18 days after treatment. A smaller number of TAA-CD8^+^ T cells were present at day 8 in control animals, but they were barely detectable at day 13 and day 18 time points, and not significantly different when compared with untreated (control) mice (Fig. 4a).

As measured on day 7, these splenic SIINFEKL^+^CD8^+^ T cells from IT-pIL12/EP-treated mice expressed significantly increased levels of the Ki-67 proliferation marker, as well as the cytotoxic T-cell marker, granzyme B (Figs. 4b, c), suggesting that this population represents effector CD8^+^ T cells (T^eff^). SIINFEKL^+^ T^eff^ cells comprised 8.8% of total splenic CD8^+^ T cells (Fig. 4c). Of note, an enrichment of both Ki-67^+^ and granzyme B^+^ CD8s were also observed in the SIINFEKL^NEG^ population in the IT-pIL12/EP mice. These results suggest that IT-pIL12/EP is driving the generation of antigen-specific CD8 T cells directed against other tumor-associated antigens, as well as the immunodominant SIINFEKL antigen.

**Fig. 5:**
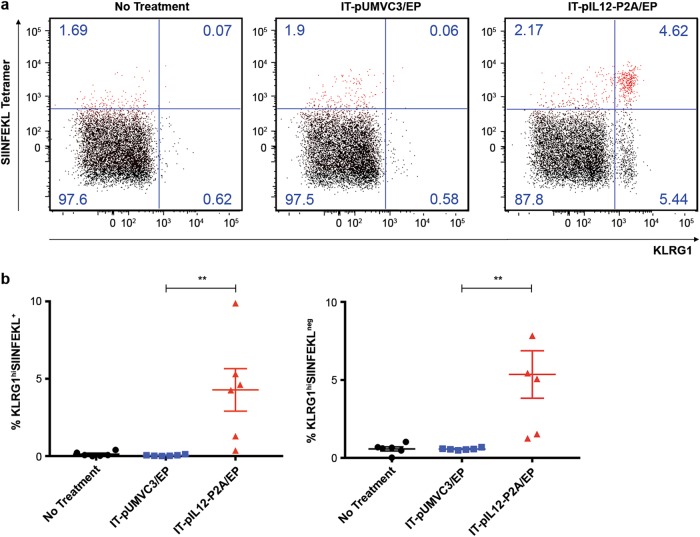
IT-pIL12/EP enriched for a KLRG1^hi^ subpopulation in splenocytes from mice bearing B16-OVA tumors. Splenocytes were isolated 8 days post EP from B16-OVA tumor-bearing mice intratumorally electroporated with pIL12-P2A or pUMVC3 or left untreated (no treatment) and analyzed by flow cytometry. **a** Dot plots for representative mice from the IT-pIL12/EP, the IT-pUMVC3/EP, and the no treatment cohorts show KLRG1 and SIINFEKL tetramer status of CD8^+^ T cells; SIINFEKL^NEG^ cells are black and SIINFEKL^+^ cells are shown in red. Prior to the analysis shown, splenocytes were gated for LIVE/CD19^NEG^/NK1.1^NEG^/CD3^+^/CD8^+^ events. **b** Scatter plots show quantification (%) of KLRG1^hi^SIINFEKL^+^ and KLRG1^hi^SIINFEKL^NEG^ CD8 T populations at day 8 for all mice (represents quantification of numbers in upper right and upper left quadrants of dot plots shown in (**a**), respectively). Both SIINFEKL^+^ and SIINFEKL^NEG^ CD8 lymphocytes were significantly enriched for KLRG1^hi^ expression in IT-pIL12/EP mice compared with empty vector/EP (*n* = 6 per cohort; **p = 0.004, one-way ANOVA). Results presented were confirmed in two other independent electroporation experiments (color figure online)

In addition, the majority of splenic SIINFEKL^+^CD8^+^ T cells from IT-pIL12/EP-treated mice had high expression of the KLRG1 cell surface marker (Fig. 5a, SIINFEKL^+^ cells shown in red). In contrast, in IT-pUMVC3/EP-treated mice, the few SIINFEKL^+^CD8^+^ T cells detected had much lower KLRG1 expression. Quantification of the percentage of SIINFEKL^+^ and SIINFEKL^NEG^ CD8 T cells at day 8 that were KLRG1^hi^ are shown in Fig. 5b. KLRG1, while ascribed various functions that are cell type and context dependent, is an effector T-cell marker [23] and known to be upregulated directly by IL-12 signaling [24]. We find that KLRG1-positive CD8 cells were mostly all granzyme B (~80%), as well as Ki-67-positive (Figure S4) thus suggesting the KLRG1-positive CD8+ T cells are potent effectors and are proliferating. Taken together, these data suggest that IT-pIL12/EP led to the significant expansion of a unique population of KLRG1^hi^CD8^+^ TAA-T^eff^ cells that persist in the periphery longer than in control animals in this model.

### IT-pIL12/EP generated a unique TIL population of KLRG1^hi^CD8^+^ T cells with diminished expression of multiple T-cell checkpoint proteins in distant, contralateral lesions

**Fig. 6:**
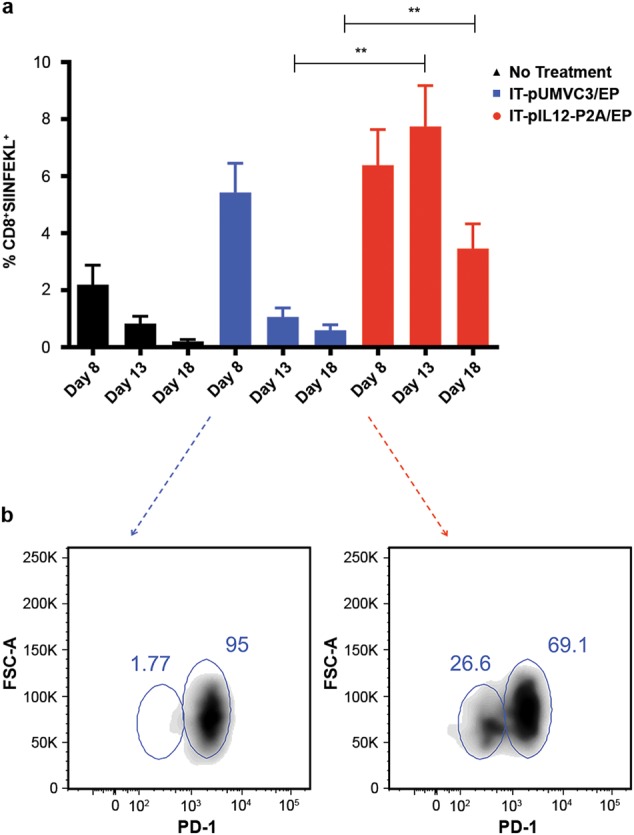
Analysis of tumor-infiltrating lymphocytes (TILs) present in untreated tumors over time; persistence of SIINFEKL^+^CD8^+^ T cells in IT-pIL12/EP-treated mice. Lymphocyte fraction of cells isolated from excised tumors were stained with SIINFEKL tetramers and other lymphocyte markers and analyzed by flow cytometry. Prior to analysis shown, tumor cells were gated for LIVE/CD19^NEG^/NK1.1^NEG^/CD3^+^/CD8^+^ events. **a** Bars in graph shown represent the mean ± SEM percentage of CD8^+^ TIL that were SIINFEKL tetramer^+^ at 8, 13, and 18 days after IT-pIL12/EP (red) or IT-pUMVC3/EP (blue) treatment of tumors on the opposite flank. Tumors from untreated mice (black) are shown for comparison; (*n* = 6 mice per cohort per time point; total *n* = 18 for each treatment group; **p = 0.0022 for day 13 and 0.0043 for day 18, Mann–Whitney test). **b** Density plots showing PD-1 staining (*x* axis) vs. forward scatter area (FSC-A; *y* axis) of SIINFEKL^+^CD8^+^CD3^+^ TIL from the untreated tumors of representative mice on day 8 from IT-pIL12/EP (right) and IT-pUMVC3/EP (left) treated cohorts to illustrate PD-1^lo^ satellite population detectable in IT-pIL12/EP-treated mice (color figure online)

TILs were isolated from the contralateral B16F10-OVA tumors and analyzed by flow cytometry on days 8, 13, and 18 post EP. IT-pIL12/EP induced an increase in the number of total CD8^+^ TIL in these distant lesions, particularly at later time points, with little effect on the levels of CD4^+^ T cells or regulatory T cells (T_reg_) (Figure S5). We focused our analysis principally on the tumor antigen-specific SIINFEKL^+^CD8^+^ TIL populations. On day 8, SIINFEKL tetramer^+^ CD8^+^ T cells were detected in the TIL from all treatment groups. Both IT-pIL12/EP and IT-pUMV3-EP-treated mice had higher levels of these cells than did the untreated control mice (Fig. 6a). However, by day 13, the SIINFEKL^+^CD8^+^ population diminished in control groups (<1% of total TIL) but persisted in IL-12-treated animals (~7%).

**Fig. 7:**
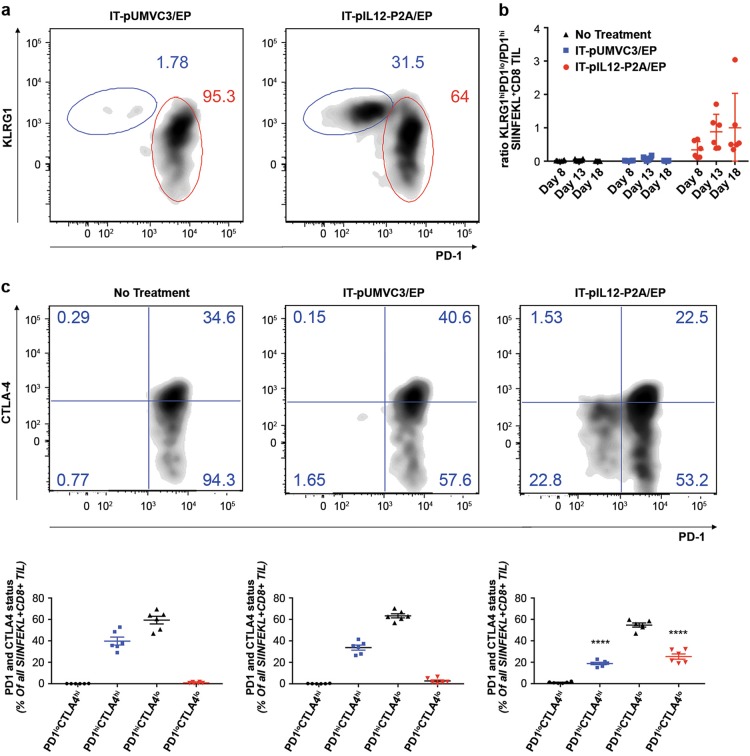
IT-pIL12/EP enriched for KLRG1^hi^CD8^+^ T cells in the contralateral tumors that were PD-1^lo^, CTLA-4^lo^. Lymphocyte fractions were isolated from excised, tumors 8, 13 and 18 days post EP from B16-OVA tumor-bearing mice, which had been treated with IT-pIL12-P2A-EP or IT-pUMVC3/EP of the tumors on the opposite flank, or left untreated (no treatment) and analyzed by flow cytometry. **a** Representative density plots show relative KLRG1 (*y* axis) and PD-1 (*x* axis) cell surface marker staining for all SIINFEKL^+^CD8^+^ TIL from IT-pIL12/EP (right panel) and IT-pUMVC3/EP (left panel) treated mice on day 18. Gating for KLRG1^hi^PD-1^lo^, and PD-1^hi^ populations are shown as blue and red ovals, respectively. Parent populations are all LIVE/CD19^NEG^/NK1.1^NEG^/CD3^+^/CD8^+^ TIL. Presence of these KLRG1^hi^PD-1^lo^ satellite populations were seen in IT-pIL12/EP-treated mice in two other independent electroporation experiments. **b** Scatter plots show the ratios of KLRG1^hi^PD-1^lo^/PD-1^hi^ in SIINFEKL^+^ CD8^+^ TIL at the time points indicated. All mice demonstrated measureable increase in this ratio at all time points measured. The numerical value for ratios of the two populations in the untreated and pUMVC3 cohorts (black and blue symbols) could not be accurately determined because the KLRG1^hi^PD-1^lo^ event count was < 50 in most all mice so statistical analysis was not done. **c** Upper panels: representative density plots for PD-1 (*x* axis) vs. CTLA-4 (*y* axis) cell surface marker expression are shown for mice with no treatment (left), IT-pUMVC3/EP (middle), and IT-pIL12/EP (right) at 18 days post EP for all TIL that were LIVE CD19^neg^NK1.1^neg^/CD3^+^/CD8^+^/SIINFEKL^+^. Lower panels: the percentage of cells from quadrants shown for representative mice shown in upper panels are graphed below each panel as a mean ± SEM for all mice; *n* = 6 per cohort per time point. PD-1 and CTLA-4 double-positive (hi) cells are shown in blue, and double-negative (lo) cells are shown in red. IT-pIL12/EP-treated mice had increased PD-1^lo^CTLA4^lo^ and decreased PD-1^hi^CTLA4^hi^ populations when compared with both cohorts of control mice that was significant (****p < 0.0001, one-way ANOVA) (color figure online)

When the SIINFEKL^+^CD8^+^ population detected in contralateral TIL from IT-pIL12/EP and IT-pUMV3-EP-treated mice at day 8 were compared for expression of PD-1, IL-12-treated animals had a distinct cluster of PD-1^lo^ cells (Fig. 6b; compare density plots from representative IL-12 vs. empty vector-treated animals). These results showed that, while both cohorts of animals had significant TAA-CD8^+^ TIL at this time point, these cells differed in their expression of the cell surface PD-1 exhaustion marker. When the relative expression of both the KLRG1 and PD-1 cell surface markers were examined together in distant tumor TIL, The SIINFEKL^+^CD8 TIL from control (no treatment and IT-pUMVC3/EP-treated) mice showed a predominant unimodal population of KLRG1^lo^PD-1^hi^ CD8^+^ T cells (only IT-pUMVC3/EP is shown; Fig. 7a; circled in red). In sharp contrast, however, TIL from the IT-pIL12/EP group exhibited a population of KLRG1^hi^PD-1^lo^ cells (blue circles), in addition to the baseline KLRG1^lo^PD-1^hi^ population seen in empty vector-treated mice. This KLRG1^hi^PD-1^lo^ population represented at least 30%; Fig. 7a) of the SIINFEKL^+^CD8^+^ TIL by day 13 and only emerged in response to IT-pIL12/EP treatment, suggesting that these TIL may be related to T^eff^ cells described previously in the spleen (see Figs. 4 and 5). The ratio of these two discrete SIINFEKL^+^CD8 TIL populations (i.e., KLRG1^hi^PD-1^lo^/PD-1^hi^) was increased in the IT-pIL12/EP mice compared with both untreated (no treatment) and IT-pUMVC3/EP control animals at all time points (Fig. 7b). Of interest, when a similar analysis was done on the SIINFEKL^NEG^CD8^+^ TIL, presumably directed against other tumor antigens, a smaller, yet significant increase in these KLRG1^hi^PD-1^lo^ TIL was seen (Figure S6). This distinct population of TIL present in IT-pIL12/EP-treated mice was detected at all three time points examined in this experiment, and was recapitulated in two other independent electroporation experiments (data not shown).

In order to better understand the significance of this low-level expression of PD-1 on these antigen-specific KLRG1^hi^ CD8 T cells, we analyzed the co-expression of CTLA-4 as high levels of PD-1 and CTLA-4 on TIL have been shown to identify exhausted CD8^+^ T cells in melanoma [25, 26]. TIL from all groups of mice demonstrated a similar uniform population of PD-1^hi^ CD8 T cells with significant percentage of high co-expression of CTLA-4, indicating the presence of a baseline exhausted tumor antigen-specific CD8 population. Only, however, in the IT-pIL12/EP-treated mice, was a distinct PD-1^lo^CTLA4^lo^ CD8 population observed (Fig. 7c), which failed to upregulate PD-1 or CTLA-4 upon infiltration into the antigen-rich microenvironment of the contralateral tumor. The percentage of T cells that were PD-1^lo^CTLA4^lo^ was significantly higher in the IT-pIL12/EP-treated group as compared with both control groups (Fig. 7c, lower panels). Expression of TIM-3 and LAG-3 were found to parallel the unexpected expression patterns of CTLA-4 and PD-1 (Figure S7), suggesting a coordinated downregulation of these checkpoint proteins in the IT-pIL12/EP emergent KLRG1^hi^PD-1^lo^ CD8^+^ TIL population.

## Discussion

In a Phase 1 clinical trial in advanced melanoma, EP-mediated transfection of plasmid-encoded IL-12 was safe and led to local necrosis and inflammation in the electroporated tumor, as well as regression of distant untreated lesions in some cases [5, 27]. Phase 2 clinical trials confirmed that IT delivery of IL-12 plasmid DNA by electroporation (Immunopulse® IL-12) induced objective responses including abscopal regressions of distant untreated lesions [27, 28]. However, only 10% of patients receiving IT-pIL12/EP show a complete therapeutic response. In a recently published paper [16], we demonstrated that improving electroporation parameters and expression of plasmid-derived IL-12p70 enhanced the systemic antitumor response with only a single treatment in mouse models. Higher IL-12 expression was achieved through both an improved plasmid design, as well as reduced voltage and increased pulse length, which led to increased IFNγ and a higher CD8^+^/ CD4^+^ T-cell ratio with the tumor. Further, a dose-response tumor regression data suggested that an increase in IL-12 expression increased efficacy [16].

Using a two-tumor murine model, we explored the mechanism of action of the new IT-pIL12/EP platform, particularly the IT-pIL12/EP-mediated perturbations of distant non-treated contralateral tumors. In the low TIL B16F10 two-tumor mouse model, regression of the treated lesion was associated with rapid induction of IL-12-regulated pathways, as well as upregulation of cytokines, chemokines and antigen presentation pathways (Fig. 2). An increased influx of lymphocytes and dramatic changes in gene expression consistent with strong inflammatory response in the distant, contralateral lesions indicated a strong abscopal response in IT-pIL12/EP-treated mice (Fig. 3).

B16F10 tumors, like most cutaneous melanomas arising in patients, have a relatively large mutational load with a correspondingly high prevalence of predicted neoantigens [29]. The poor immunogenicity of the B16F10 mouse tumor model has been attributed to a lack of effective antigen presentation due to epigenetic suppression of APM, including MHC Class I, MHC Class II and Tap-1 [30]. IFN-γ exposure is known to potently reverse this methylation-dependent epigenetic suppression of APM [31]. In this study, we show that IT-pIL12/EP led to an increase in IFN-γ in treated tumors, similar to previous reports [7, 14], and further analyze IFN-γ-responsive genes involved in antigen processing and presentation in treated lesions (Fig. 2). Previously, Restifo and colleagues [32] elegantly demonstrated that IL-12 delivered IT to B16F10 tumors using transgenic pmel CD8 T cells led to similar IFN-γ upregulation of APM and converted abundant IT myeloid-derived suppressor cells into cross-presenting APCs. Thus, IT-pIL12/EP may be acting like an in situ vaccine [15], resulting in the induction of immunogenic cell death, uptake of tumor-associated antigens by APCs in the appropriate pro-inflammatory milieu to enhance immunogenic cross-presentation.

Using a two-tumor model with a B16F10 cell line variant expressing a traceable antigen, we show that IT-pIL12/EP resulted in dissemination of tumor antigen directed (SIINFEKL tetramer positive) CD8 T cells in the spleen that persisted for up to 18 days after a single treatment (Fig. 4a). In addition, a significant enrichment of splenic SIINFEKL^+^ CD8 T cells that expressed effector T-cell markers (GRZMB^+^ or Ki-67^+^) was detected (Figs. 4b, c). Furthermore, the IT-pIL12/EP-dependent SIINFEKL^+^ T^eff^ population in the spleen was found to be predominantly KLRG1^hi^ (Fig. 5 and S4). In addition to the SIINFEKL^+^ splenocytes, we found a significant number of SIINFEKL^NEG^ granzyme B^+^ (Fig. 4) and SIINFEKL^NEG^ KLRG1^+^ (Fig. 5) CD8^+^ T cells suggesting that IT-pIL12/EP also induced effector T-cell directed against other endogenous B16F10 antigens [29]. We also note a similar SIINFEKL^NEG^ population in contralateral tumor TIL (Figure S6).

In a previous study, Sin and co-workers demonstrated that IT electroporation in B16F10 tumors induced systemic CD8 T-cell responses directed against a known endogenous B16F10 antigen (Trp2180–188 epitope) and generated therapeutic effects against distant, contralateral tumors [7]. They further show that pretreatment of mice with anti-CD8 antibodies significantly reduced the efficacy of the IL-12/EP therapy thus demonstrating the importance of the CD8 T-cell response. Our study complements and extends these observations by further characterizing TAA-CD8^+^ cells within the, distant, contralateral tumors. Taken together with the work of Sin et al., our studies strongly suggest that IL-12 treatment generates and disseminates TAA-CD8^+^ cells that participate in the antitumor response in distant, contralateral tumors.

Strong IL-12 signaling during T-cell priming has been implicated in the generation of KLRG1^+^ effector CD8 T cells through upregulation of the transcription factor T-bet [24] and can lead to increased longevity of these cells in vivo upon adoptive transfer [33–35]. Other studies attribute optimal differentiation into effector CD8 T cells to Blimp-1 and T-bet, both IL-12-driven nuclear transcription factors [21]. Consistent with these reports, transcripts for both *T-bet* and *Blimp-1* genes were upregulated in the IT-pIL12/EP-treated lesions (Fig. 2) and significant KLRG1 expression on CD8^+^ T cells was only present in IT-pIL12/EP mice, Furthermore, KLRG1^hi^CD8 T cells were detected in the spleen for up to 18 days. In our model, KLRG1 expression seems to serve as a useful phenotypic biomarker of successful T cells priming with IL-12 in situ in electroporated tumors.

IT-pIL12/EP treatment resulted in the generation of both KLRG1^hi^CD127^NEG^, as well as a population of “double-positive” KLRG1^hi^CD127^+^ CD8 T cells (data not shown). The significance of these phenotypes in the context of cancer immunotherapy remains unresolved, but similar KLRG1^+^CD127^NEG^ and KLRG1^+^CD127^+^ CD8s effector cell populations have been identified in various viral infection models and named short-lived effector cells (SLECs) and double-positive effector cells (DPECs), respectively [24]. SLECs, as the name connotes, are thought to be highly potent, terminally differentiated cytotoxic T cells, which have limited proliferative potential and do not contribute to the formation of long-term memory precursor populations [36]. There is evidence for KLRG1 as not only an effector T-cell marker, but also one for terminal differentiation and senescence in T cells, and KLRG1 has been shown to act as a co-receptor to inhibit cytotoxic function of T cells toward E-cadherin-expressing cells [37]. Currently, it remains unclear whether the KLRG1^hi^ CD8’s produced by IT-pIL12/EP represent classical SLECs and DPECs or have more “promiscuous CTL traits” as previously ascribed to IL-12-primed CD8’s [23, 38].

Flow cytometric analysis of the baseline SIINFEKL^+^ CD8 population detected in the distant, untreated B16F10-OVA tumors from control animals (IT-pUMVC3/EP) revealed a single major PD-1^hi^ population, which based on PD-1^hi^ status and co-expression of other T-cell checkpoint receptors (CTLA-4, LAG-3, TIM3) likely represents CD8 T cells in various stages of evolving exhaustion/dysfunction. We would expect to observe a PD-1^hi^ phenotype on infiltrating SIINFEKL-reactive KLRG1^hi^ CD8 T cells due to either (1) activation upon re-encounter with antigen (i.e., OVA) or (2) exhaustion due to re-encounter with antigen in the presence of increased PD-L1 expression. Contrary to this expectation, however, many of the SIINFEKL^+^ KLRG1^hi^ CD8 T cells in the IT-pIL12/EP group express distinctly low, but non-negative, levels of PD-1, as well as diminished expression of multiple T-cell other checkpoint proteins, including CTLA-4, Lag-3, and Tim3 (Fig. 7 and S7). Although the mechanism of this coordinated downregulation of checkpoint molecules remains to be established, Blimp-1 has been shown to be capable of physically displacing Nuclear factor of activated T-cells  (NFAT-1C) from the PD-1 promoter, leading to decreased transcription of PD-1 [39]. Similarly, as NFAT-binding sites are present in the promoter regions of CTLA-4, Tim-3, and Lag-3 [40, 41], IL-12-mediated overexpression of Blimp-1 during T-cell priming in electroporated tumors may represent the common mechanism. At this juncture, we do not know if these KLRG1^hi^PD-1^lo^ CD8 TIL increase their PD-1 expression over time as they persist in the tumor, eventually converting to a PD-1^hi^ exhausted state.

It is interesting to consider whether the IL-12-dependent downregulation of PD-1 (and other checkpoints) on the KLRG1^hi^ CD8 TIL plays a role in “armoring” these particular effector CD8^+^ T cells against T-cell checkpoints, contributing to their potent antitumor effects. Clearly, a paper by Gerner et al. suggests that this may, in fact, be the case [34]. These authors studied the antitumor effect of adoptive transfer of CD8^+^ T cells primed in the presence of either IL-12 or IFN-α in the B16F10 model. OT-1 T cells primed with K^b^-SIINFEKL together with CD80 in the presence of IL-12 (compared with IFN-α) are more effective antitumor CTL because they are programmed during the priming phase in some manner to limit their expression of PD-1 upon re-encountering antigen in the TME. Thus, anti-PD-L1 blockade fails to improve the antitumor effects of IL-12-primed OT-1 cells, whereas it rescues IFN-α-primed OT-1 CTL activity to the level of IL-12-primed cells. Other studies confirm enhanced antitumor activity of PD-1^lo^ CD8 effector T cells compared with PD-1^hi^ [42-44].

Here, we provide evidence that localized IT IL-12 expression in ELs led to the generation and dissemination of TAA-KLRG1^hi^CD8^+^ effector T cells in both the spleen and distant contralateral TILs. Interestingly, a significant abscopal effect was observed in the contralateral tumor despite induction of adaptive resistance (characterized by IFN-γ-responsive genes including PD-L1). We hypothesize that this potent antitumor effect on the distant, contralateral tumors may be orchestrated, at least in part, by highly activated KLRG1^hi^CD8^+^ T cells that are able to downregulate expression of “checkpoint proteins” in a coordinated manner and thus be transiently resistant to multiple immune checkpoints, contributing to their fitness and longevity as antitumor CTL. Although other IL-12-responsive cells likely play a contributing role in systemic tumor immunity generated with IT-pIL12/EP, we propose this KLRG1^hi^CD8^+^ T cells as a prominent mechanism of the abscopal effects of local IL-12 delivery in mice.

Fallon and co-workers have recently demonstrated that systemic IL-12 therapy (targeted to necrotic regions on the tumor) can synergize with anti-PD-L-1 checkpoint therapy in a mouse model [45]. In melanoma patients, who received IT electroporation-mediated IL-12 (Immunopulse® IL-12) and who subsequently received anti-PD-1 blockade were observed to have higher-than-expected PD-1 response rates [46, 47]. Although this study was retrospective, it suggests that, in at least some patients, IL-12 may have primed a subclinical antitumor immune response that was stalled until an anti-PD-1 agent was administered. Based on these data, the clinical benefit of IT electroporation of IL-12 in combination with pembrolizumab is being evaluated in advanced melanoma (NCT02493361; NCT03132675).

## Methods

### Mice, tumor cell lines, contralateral model

Female C57Bl/6J mice, 6–8 weeks of age (Jackson Laboratories) were housed in accordance with AALAM guidelines. B16F10 melanoma cells (CRL-6475; ATCC) were cultured with McCoy’s 5A medium (2 mM l-glutamine) with 10% fetal bovine serum (FBS) and 50 μg/ml gentamicin. Murine B16F10-OVA melanoma cell lines (a gift from Heat Biologics) were established by transfecting B16F10 cells with full-length ovalbumin and selecting for stable transformants [48], and were cultured in Iscove's Modified Dulbecco's Medium (IMDM) + 10% FBS + 2 mg/ml G418. Cells were harvested with 0.25% trypsin and re-suspended in Hank’s balanced salt solution. Anesthetized mice were subcutaneously injected with 1 million cells and 0.25 million cells each in a total volume of 0.1 ml into the right and left flank of each mouse, respectively. Tumor growth was monitored by digital caliper measurements. Tumor volume (*V*_*T*_) was calculated using he formula *V*_*T*_ = *a*^2^× b/2, where *a* = smallest diameter and *b* = perpendicular diameter. Mice with tumors ranging from 100 to 160 mm^3^ (right) and 30 to 35 mm^3^ (left) were randomized and divided into treatment groups. This protocol was used as a standard model to test simultaneously for the effect on the EL and contralateral lesion (CL). Tumor volumes were measured twice weekly. Mice were euthanized when the total tumor burden reached 1500 mm^3^.

### Plasmids (pIL12-P2A and pUMVC3)

Mouse IL-12p35 and p40 gBlock DNA fragments were obtained from Integrated DNA Technologies, Inc. (Coralville, IA) and cloned into pUMVC3 plasmid (Aldveron, Fargo, ND) separated by a transcription modifier sequence, P2A (Burkart et al. [16] and supplemental methods).

### IT treatment

Mice were anesthetized with 3% isoflurane for treatment. In all, 50 μg of circular plasmid DNA, diluted to 1 μg/μl in sterile 0.9% saline, was injected centrally into primary tumors using a 1 ml syringe with a 26-gauge needle. EP followed immediately after plasmid DNA injection, using a BTX ECM 830 square wave generator (Harvard Apparatus) or GENESIS square wave generator (OncoSec Medical Incorporated) to deliver eight unidirectional pulses (400 V/cm, 10 ms) using a four-needle electrode array.

### Tumor lysis for protein extraction and cytokine analysis by ELISA

Forty-eight hours after IT-EP, tumors were dissected from mice and frozen in liquid nitrogen. In all, 300 μl of tumor lysis buffer (50 mM TRIS pH 7.5, 150 mM NaCl, 1 mM EDTA, 0.5% Triton X-100, 1× Protease inhibitor cocktail) was added to the frozen tumor and homogenized on ice for 30 s (LabGen 710 homogenizer). Lysates were transferred to 1.5 ml centrifuge tube and spun at 10,000 × *g* for 10 min at 4 °C. Supernatants were centrifuged and transferred to a new tube; this step was repeated three times. Tumor extracts were frozen at –80 °C or analyzed immediately according to manufacturer’s instruction (mIL-12p70 DuoSet ELISA kit, DY419 and mIFNγ DuoSet ELISA kit, DY485). The P2A ELISA was modified from a commercial mouse IL-12p70 ELISA (details in supplemental methods).

### RNA extraction and gene expression analysis

Tumor tissue was harvested from mice using a scalpel and flash frozen in liquid nitrogen. Tissues were weighed, Trizol (Thermo Fisher Scientific, Waltham, MA) was added and tissue was homogenized using a probe homogenizer on ice. RNA was extracted from Trizol using manufacturer’s instructions. Contaminating DNA was removed with DNase (Thermo Fisher, cat no.: EN0525). Gene expression profiling was performed using NanoString^®^ technology. (nCounter code sets; details in supplemental methods).

### Histology and immunohistochemistry

Tumors were fixed in 10% neutral buffered formalin and embedded in paraffin according to standard procedures. Five micron sections were stained with H&E to assess tissue and cellular morphology. Immunohistochemistry to detect CD8a was performed using a polyclonal rat antibody (14-0808-82, eBiosciences; details in supplemental methods).

### Splenic lymphocytes and TIL isolation, staining for flow cytometric analysis

Spleen cells were isolated by pressing spleens through a 70 μm filter, followed by red blood cell lysis (RBC lysis buffer, VWR, 420301OBL). Tumors were dissociated using Gentle-MACS (Miltenyi tumor dissociation kit 130-096-730, C-tubes, 130-093-237) and homogenized using a Miltenyi gentleMACS™ Octo Dissociator with Heaters (130-096-427). Both tumor and spleen cell suspensions were fractionated with lympholyte-M (Cedarlane CL5035) and lymphocyte layers were washed and re-suspended in phosphate-buffered saline containing 2% FBS and 1 mM EDTA with 1 × /mL Fc block (eBioscience 14-0161-85) on ice. In 96-well plates, cells were mixed with a solution of SIINFEKL or control tetramer (MBL T03002 or T03000, TS-M008-2), according to the manufacturer's instruction, and stained with antibody cocktails (details in supplemental methods). Data analysis was done using FlowJo software (TreeStar).

## Electronic supplementary material

Supplemental materials and figure legends (clean)

All supplemental figures

Supplemental materials and figure legends (marked up)

## Data Availability

The datasets supporting the conclusions of this article are included within the article.
