# Supplementary material for: Characterization of abscopal effects of intratumoral electroporation-mediated IL-12 gene therapy
Source: Gene Ther. 2018 Oct 15;26(1):1–15. doi: 10.1038/s41434-018-0044-5 (PMC6514882; doi:10.1038/s41434-018-0044-5)
Supplement: Supplementary file 1 — Supplemental materials and figure legends (clean) [file 41434_2018_44_MOESM1_ESM.docx]

**Supplemental Figure Legends**

**Figure S1: IT-pIL12/EP led to increased infiltration of lymphocytes and activation of INF-γ pathways in treated tumors**. **(a)** Representative images of electroporated tumors at day 7 stained with H&E are shown from tumors electroporated with empty vector (pUMVC3, **left panels**), and pIL12-P2A (**right panels**). Short arrows: Extensive pleomorphic inflammatory infiltrate; long arrows: region of extensive tumor necrosis, hemorrhage, melanin pigment. Low power scale bar = 500 microns. High power scale bar = 50 microns. **(b)** Induction in gene expression indicative of immune infiltrates and **(c)** IFN-γ responsive genes, both expressed as a fold change over no treatment levels at 7 days post EP as assessed by NanoString^®^ nCounter technology (All genes presented were statistically different from controls; p<0.05, Holm-Sidak method). The dotted line represents a 2-fold increase over untreated tumor controls.

**Figure S2: IT-pIL12/EP led to early activation of pathways involved in productive immune response.** Enrichment of genes involved in the **(a)** JAK/STAT pathway, **(b)** cytokine and chemokine mediated response, **(c)** innate immune response and **(d)** inflammation are shown for treated tumors at 3 days post EP, represented as ‘Pathway scores’ (Assessed by NanoString^®^ nCounter technology), as determined by nSolver data analysis software. Pathway scores follow the assumptions of equal variance and normal distribution of t scores. The pathway scores in IL12-P2A group were significantly higher than the pUMVC3 and untreated group (p <0.0001, One-way ANOVA).

**Figure S3. Intratumoral electroporation of pIL12-P2A results in detectable plasmid-derived IL-12 protein in treated but not distant, untreated tumors.** ELISA for a P2A epitope present on the p35 subunit was used to selectively measure plasmid-derived IL-12P2A protein in treated and contralateral (untreated) tumor lysates from mice bearing B16F10 tumors 48hrs post EP (n=3). This epitope is created as a result of a small peptide left upon exon skipping during protein translation (see Burkhart et al. Reference 16). Data are presented as ng protein per 1mg total tumor lysate. IL12P2A protein was detectable in all tumors treated with IT-pIL12P2A/EP and undetectable in any of the contralateral (untreated) tumors or in any tumors from IT-pUMVC3/EP treated animals.

**Figure S4: TAA-KLRG1^hi^CD8^+^ splenocytes from IT-pIL12/EP treated mice demonstrated enhanced expression for Ki-67 and Granzyme B.** Splenocytes were isolated 7 days post EP from B16F10-OVA tumor bearing mice left untreated or intratumorally electroporated with pIL12-P2A or pUMVC3 control and analyzed by flow cytometry. Splenocytes were gated as follows prior to analysis: LIVE, CD19^NEG^NK1.1^NEG^/CD3^+^CD8^+^ SIINFEKL^+^ cells **(a)** Representative dot plot from one mouse in the pIL12-P2A cohort shows gating for granzyme B^+^KLRG1^+^CD8^+^ and granzyme B^-^KLRG1^+^CD8^+^ T cells (**left panel**). Scatter plot shows quantification (%) of granzyme B^+^ KLRG1^+^ and granzyme B^-^KLRG1^+^CD8^+^ T cells from all mice (**right panel**, n=5, p=0.007, T test, Mann-Whitney) **(b)** Representative dot plot from one mouse in the pIL12-P2A cohort shows gating for Ki67^+^KLRG1^+^CD8^+^ and Ki67^-^KLRG1^+^CD8^+^ T cells **(left panels**). Scatter plots show quantification (%) of Ki67^+^KLRG1^+^ cells and Ki67^-^ KLRG1^+^ CD8^+^ T cells from all mice (**right panels**, n=5, p=0.02, T test, Mann-Whitney).

**Figure S5: Tumor infiltrating lymphocytes (TIL) present in untreated tumors over time; persistence of CD8^+^ T cells in IT-pIL12/EP treated mice.** All cells isolated from excised tumors were stained with lymphocyte markers and analyzed by flow cytometry**.** Prior to analysis shown, tumor cells were gated for LIVE/CD19^NEG^/NK1.1^NEG^/CD3^+^events. Bars represent the mean +/- SEM percentage of CD3+ TIL that are CD8^+^ (**top**), CD4^+^ (**middle**), and CD4^+^CD25^+^FoxP3^+^ (**bottom**) at 8, 13, and 18 days after IT-pIL12/EP (**red**), IT-pUMVC3/EP (**blue**) treatment of tumors on the opposite flank**.** Tumors from untreated mice are shown for comparison (black)**;** n=6 mice per cohort per time point (total n=18 for each treatment group); CD8^+^, **p=0.0087 for Day 13 and Day 18 CD4^+^, **p=0.0043 for Day 18, Mann-Whitney tests.

**Figure S6: KLRG1 and PD1 expression in SIINFEKL^NEG^ contralateral TIL.** Lymphocyte fractions were isolated from excised, untreated tumors 8, 13 and 18 days post EP from B16F10-OVA tumor bearing mice electroporated with IT-pIL12-P2A or IT-pUMVC3 in tumors on the opposite flank or left untreated and analyzed by flow cytometry. **(a)** Representative density plots show the relative KLRG1 (y-axis) and PD-1 (x-axis) cell surface marker staining for all SIINFEKL^NEG^ CD8^+^ TIL from IT-pIL12/EP (**right panel**) and IT-pUMVC3/EP (**left panel**) treated mice on Day 18. Gating for KLRG1^hi^PD1^lo^, and PD1^hi^ populations are shown as blue and red ovals, respectively. Parent populations are all LIVE/CD19^NEG^/NK1.1^NEG^/CD3^+^/CD8^+^ TIL. **(b)** Scatter plots show the ratios of KLRG1^hi^PD1^lo^/PD1^hi^ in SIINFEKL^NEG^ CD8^+^ TIL at the time points indicated. The numerical value for ratios of the two populations in the untreated and pUMVC3 cohorts (black and blue symbols) could not be accurately determined because the KLRG1^+^PD1^lo^ event count was <50 in most all mice so statistical analysis was not done.

**Figure S7: IT-pIL12/EP treatment enriches for SIINFEKL^+^CD8^+^ T cells in the contralateral tumors that express low levels of PD-1, Tim3, and Lag3.** Representative density plots for PD-1 (x-axis) *vs.* Tim3 (left panel), or LAG3 (right panel) (y-axis) cell-surface marker expression on SIINFEKL^+^CD8^+^ TIL from distant tumors at the Day 18 time point post-EP are shown for mice with no treatment **(top)**, IT-pUMVC3/EP **(middle)** and IT-pIL12/EP **(bottom).** To the right of each set of density plots, the percentage of cells from quadrants are graphed as a mean +/- SEM for all mice; n=6 per cohort. Double positive cells are shown in blue, and double negative cells are shown in red for PD-1 and other paired exhaustion markers in each graph. Double positive and double negative populations were found to be significantly different in IT-pIL12P2A/EP treated mice as compared to both controls (**p=0.032, ***p=0.0005, ****p<0.0001, two-way ANOVA). The profiles looked similar at Day 8 and Day 13 time points, with IT-pIL12/EP treated animals having significantly more PD1^lo^Tim3^lo^ and PD1^lo^Lag3^lo^ tumor antigen-specific T cells in untreated tumor TIL. Results with the PD-1 and Lag3 exhaustion markers were repeated in two other independent electroporation experiments.

**Supplementary methods**

**P2A ELISA**

The P2A ELISA was modified from the commercial mouse DuoSet IL-12p70 ELISA (R&D DY419).  After analyte capture with the IL-12 p70 antibody, detection was performed using an anti-2A peptide antibody (Millipore, cat. #ABS31; 1:10,000 dilution) followed by incubation with a peroxidase-conjugated donkey anti-rabbit antibody (Jackson ImmunoResearch, cat. #711-035-152; 1:20,000 dilution).  Colorimetric detection was performed using the kit reagents and absorbance (abs) read on a Spectromax plate reader .  A standard curve was generated from dilutions of HEK293 cell extracts after transfection with mouse IL-12p70-P2A (Burkart et al) ranging from 156.4 – 10,000 pg/mL.

**Plasmid cloning (pIL12P2A)**

Mouse IL-12p35 and p40 gBlock DNA fragments based on accession numbers (NM_001159424

and NM_001303244, respectively) were obtained from Integrated DNA Technologies, Inc. (Coralville, IA) and cloned into pUMVC3 plasmid (Aldveron, Fargo, ND) separated by a transcription modifier sequence, P2A. Briefly, the IL-12 p35 gBlock was PCR amplified with primers that inserted 5’ Xba1 and 3’ Not1 sites, and the IL-12p40 gBlock was PCR amplified with primers that inserted 5’ BamH1 and 3’ BgIII sites. Both IL-12 subunits were restriction enzyme digested and ligated together separated by an in-frame P2A sequence, generating mIL-12p35-P2A-mIL-12p40/pUMVC3 (pIL12P2A). Positives clones were identified via restriction enzyme digests and verified with DNA sequencing.

**Nanostring® gene expression analysis**

50ng of total RNA was hybridized at 96°C overnight with the nCounter® (Mouse immune ‘v1’ Expression Panel, NanoString® Technologies). This panel profiles 561 immunology-related mouse gene as well as 15 housekeeping genes. Hybridized samples were then digitally analyzed for frequency of each RNA species using the nCounter SPRINT™ profiler. Raw mRNA abundance frequencies were analyzed using the nSolver™ analysis software 2.5 pack. Normalization factors were derived from the geometric mean of housekeeping genes, after QC with built in negative and positive controls. Heat maps were generated using nSolver™ analysis software 2.5 pack. Fold-change in expression levels was calculated by dividing individual expression values (normalized reads) for each mouse by the mean of values from untreated tumors.

**Staining of splenocytes and tumor TIL for flow cytometry**

For data presented in Figure 4, antibody staining was performed with one panel containing antibodies against CD3, CD4, CD19, NK1.1, KLRG1, CD127 (BD Biosciences 7065929, 564922, 561737, 560718, 740279, 564175); CD8 (MBL D271-4); Ki67 and granzyme B (Biolegend 652419, 515408) and vital stain (BD Biosciences 565388) and for Figures 3, 5, 6 and 7 with two flow panels containing antibodies against CD3, CD19, NK1.1, CD4, CD8a, CD44, CTLA4, Tim3, PD1, KLRG1, CD127 (Biolegend 100225, 115546, 108738, 100451, 100742, 103041, 106316, 134003, 135228, 138418, 135040), Lag3 (eBioscience 48-2231-82) and vital stain (Thermo-Fisher L-34966). Cells were either analyzed immediately or fixed in PFB with 1% paraformaldehyde for 1 minute on ice, and stored in the dark at 4’C until acquisition. Samples were acquired on an LSR II flow cytometer (Beckman) or BD FORTESSA X20.

**Histologic and Immunohistochemical staining**

Deparaffinized 5 micron sections were treated with 3% H_2_0_2_ then subjected to heat-induced antigen retrieval in citrate buffer at pH 6.0 and a temperature of 100°C on the Leica Bond autostainer. The primary antibody was diluted to 1:100 detected with a rabbit anti-rat secondary (Rockland 712-4126 at 1:500 dilution). The secondary antibody was detected using an HRP-containing polymer (Powervision-Rbt-HRP, Leica PV6119) followed by incubation with 3,3′-Diaminobenzidine (DAB). Hematoxylin was used as a counterstain. Images were captured using the Aperio VERSA digital scanner.

**Statistical analysis**

All data are expressed as mean ± SEM unless otherwise indicated. Statistical analyses were performed using non-parametric Mann-Whitney U-tests or Kruskal-Wallis analyses with multiple comparison Dunn's post test using Prism software (GraphPad Software Inc. San Diego, CA). The number of animals analyzed is shown in figure legends. Differences between groups were considered to be statistically significant as follows: (*) p < 0.05, (**) p <0.01, and (***) p<0.001. In some cases exact p values are noted in the figure legends. For flow cytometry data, exact fractions or percentages of populations that comprised <50 events were considered not determinable (ND), except when analyzed from within quadrants using a 2-way ANOVA, with Tukey’s multiple comparison post test (i.e Figures 7c and S7). For all animal experiments, in accordance with the Animal Welfare Act the number of animals per treatment group was reduced to the lowest possible number to still achieve an estimated statistical power of 80%. The animal technician measuring the tumors was blinded to the study.
