# Supplementary figures and images for: Characterization of abscopal effects of intratumoral electroporation-mediated IL-12 gene therapy
Source: Gene Ther. 2018 Oct 15;26(1):1–15. doi: 10.1038/s41434-018-0044-5 (PMC6514882; doi:10.1038/s41434-018-0044-5)

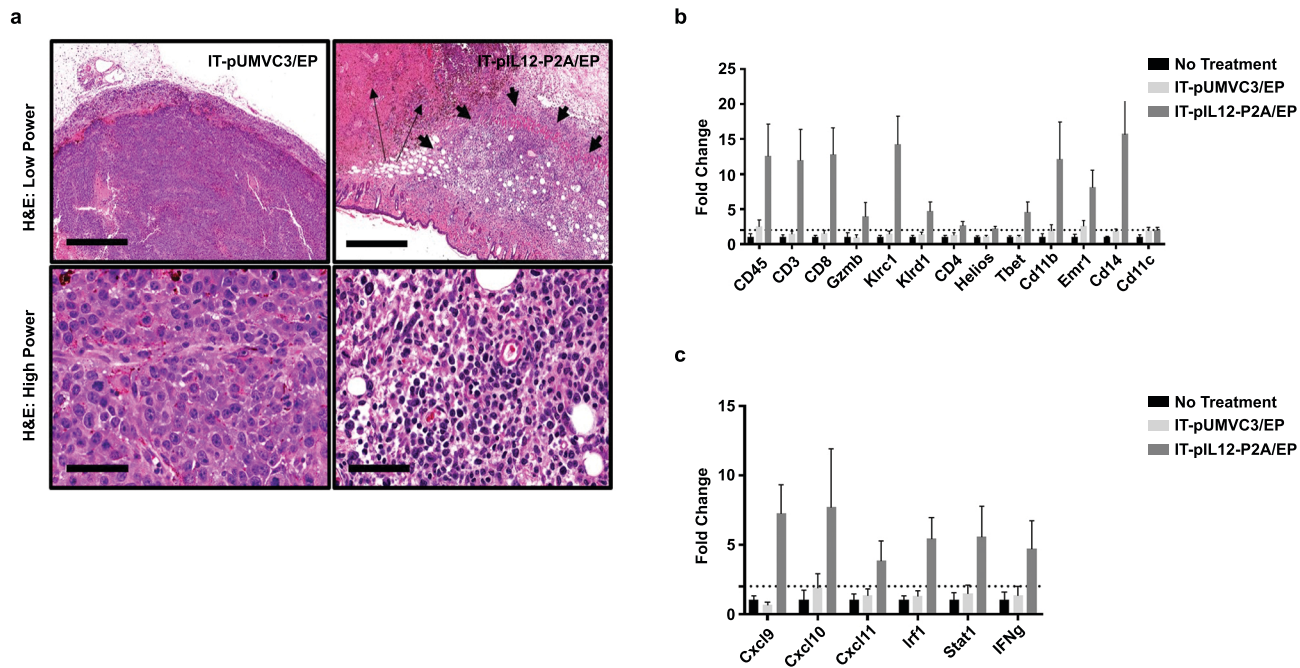

a

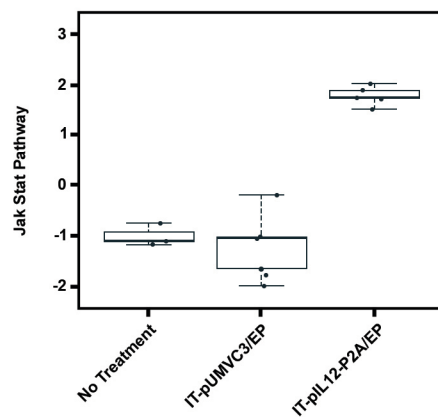

b

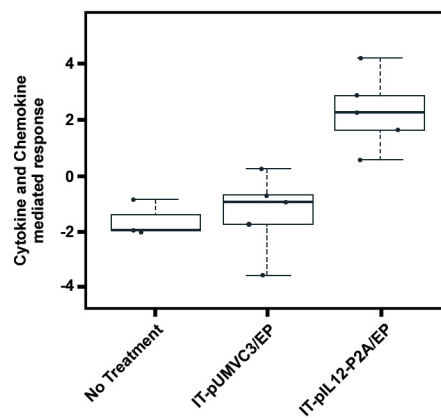

c

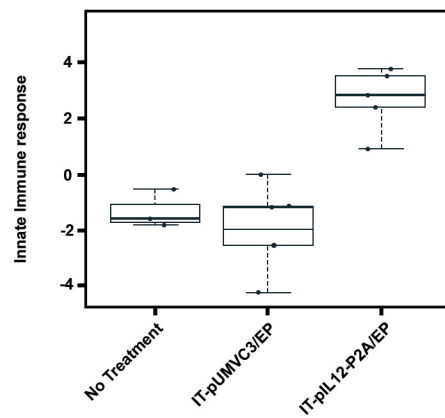

d

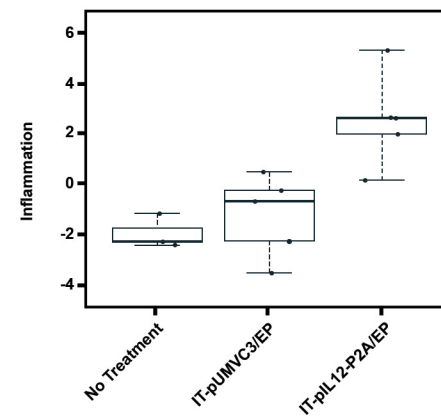

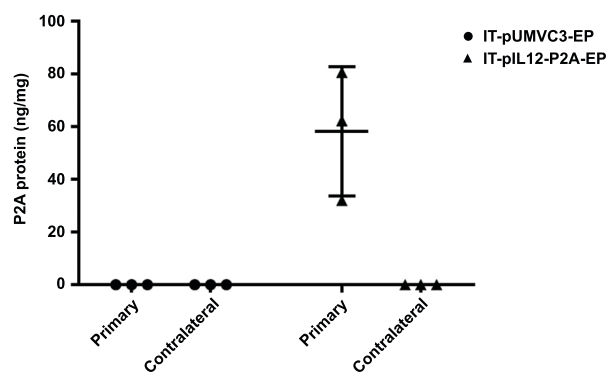

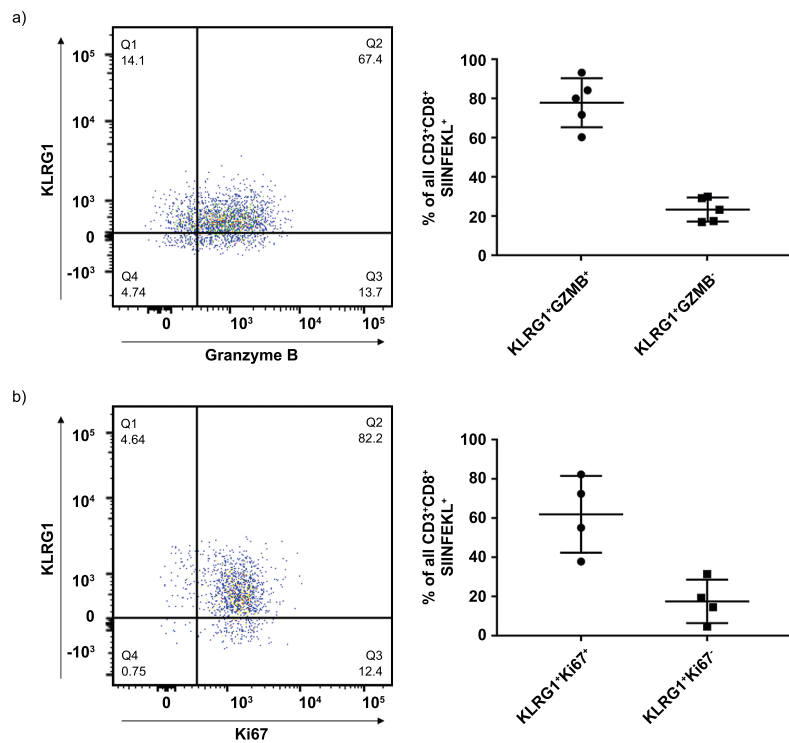

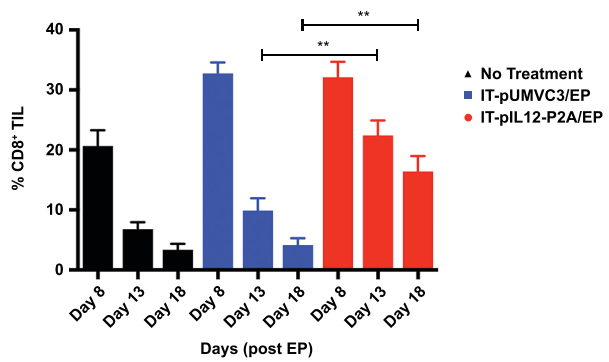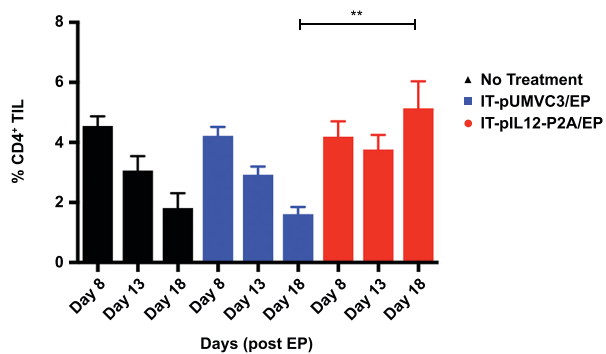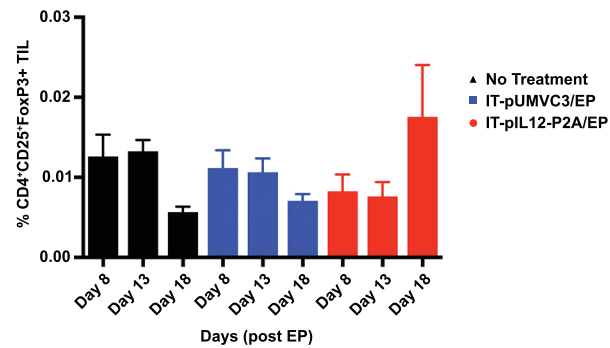

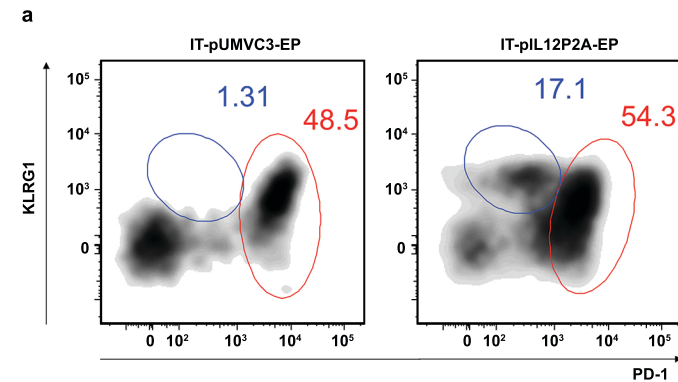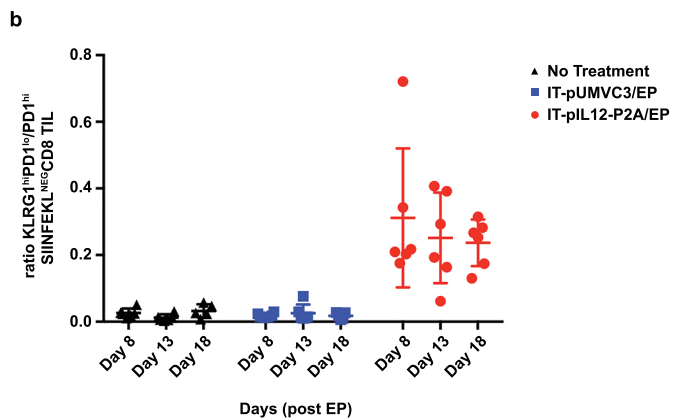

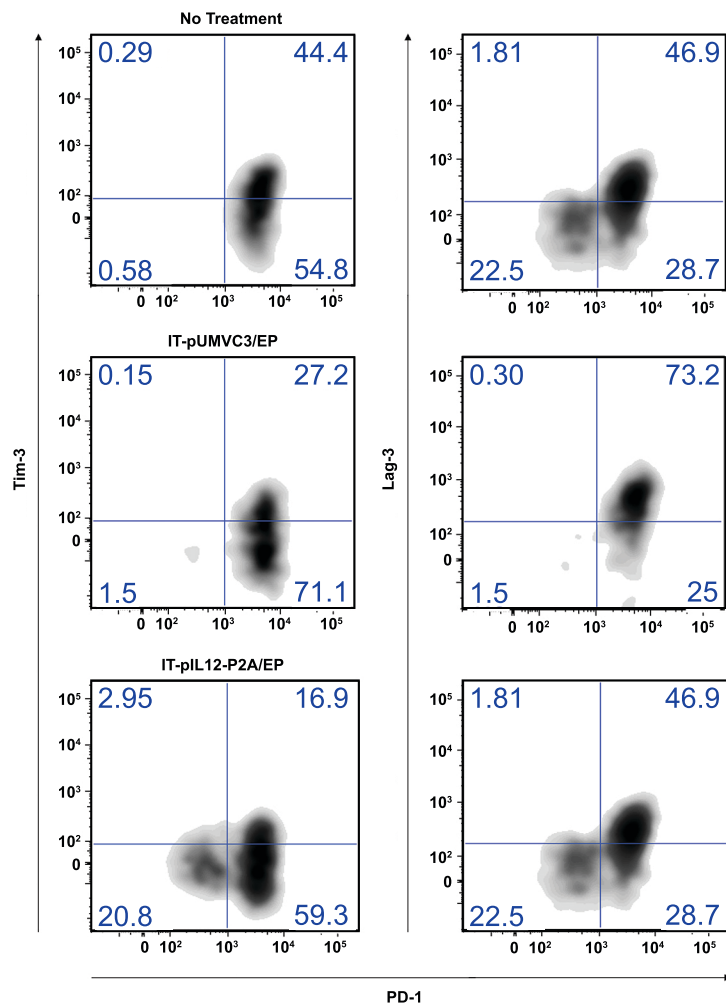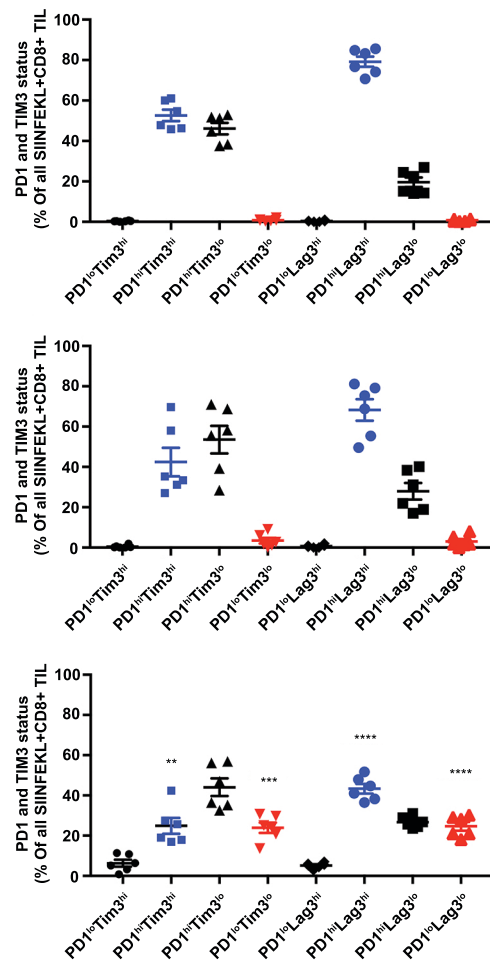

Supplement: Supplementary file 2 — All supplemental figures [file 41434_2018_44_MOESM2_ESM.pdf]
